# The Genus Homo

**Published:** 1881-03

**Authors:** 


### THE GENUS HOMO.

The average weight of an adult man is 140 pounds 6 ounces.

The average weight of a skeleton is 14 pounds.

The number of bones, 200.

The skeleton measures one inch less than the height of the living man.

The average weight of the brain of a man is 3½ pounds ; of a woman, 2 pounds 11 ounces.

The brain of a man exceeds twice that of any other animal.

The average height of an Englishman is 5 feet 9 inches ; of a Frenchman, 5 feet 4 inches, and of a Belgian, 5 feet 6¾ inches.

The average weight of an Englishman is 150 pounds ; of a Frenchman, 136 pounds, and of a Belgian, 140 pounds.

The average number of teeth is 31.

A man breathes about 20 times in a minute, or 1,200 times in an hour.

A man breathes about eighteen pints of air in a minute, or upwards of seven hogsheads in a day.

A man gives off 4.08 per cent. carbonic gas of the air he respires; respires 10,666 cubic feet of carbonic acid gas in 24 hours ; consumes 10,667 cubic feet of oxygen in 24 hours, equal to 125 cubic inches of common air.

A man annually contributes to vegetation 124 pounds of carbon.

The average of the pulse in infancy is 120 per minute ; in manhood, 80 ; at sixty years, 60. The pulse of females is more frequent than that of males.

The weight of the circulating blood is about 28 pounds.

The heart beats 75 times in a minute ; sends nearly 10 pounds of blood through the veins and arteries each beat ; makes four beats while we breathe once.

540 pounds, or 1 hogshead 1¼ pints, of blood pass through the heart in one hour.

12,000 pounds, or 24 hogsheads 4 gallons, or 10,782½ pints, pass through the heart in 24 hours.

1,000 ounces of blood pass through the kidneys in one hour.

174,000,000 holes or cells are in the lungs, which would cover a surface thirty times greater than the human body.

---

I LOOK upon indolence as a sort of suicide, for the man is effectually destroyed, though the appetite of the brute may survive.—*Chesterfield.*

### PURCHASED BEAUTY.

A lady who has patronized an institution devoted to beautifying the female face and figure, says the result is simply this : No woman can paint without detection. Devotees of fashion may just as well abandon the contrary opinion. I looked into the mirror on getting out of the chair, and hardly recognized myself. My face was greatly changed. My eyes shone, my cheeks glowed, and there was a brightness and piquancy that had not been there when I entered. But this, mind you, was in a somewhat dimly-lighted room, where the work was softened and shaded. Ten minutes afterward I met myself in a street mirror, under the full glare of a noonday sun. Well, I was simply disgusted. The painted surface looked no more like human skin than it did like sole leather ; the black around my eyes was like strokes of char-coal ; my lips had the unnatural red of scarlet ink. I walked up to the glass and viewed my artificial countenance with a feeling of repulsion. It reminded me of some execrable portrait done in water colors. I hurried into a store and bought a veil, with which I covered the beautification. Then I went straightway home, and scrubbed my face until every trace of foreign substance was gone. My experience convinced me of the utter folly of paint as a beautifier, for by no possibility can it be put on without showing exactly what it is. Dry powder, and mighty little of that, is all that I advise any woman to put on her face. If nature has not imparted beauty of complexion, there is no use trying to make up the deficiency by artifice. It is far better to turn our ingenuity toward wearing our hair becomingly, for in that direction a great deal of comeliness may be commanded. But let pigments alone, unless you are content to be pretty in a ghastly kind of way, and at the sacrifice of all outward indications of warm flesh and blood.

---

An old Vermont farmer came home drunk the other night, and became the victim of an irrepressible desire to get still drunker. So he thought he would bring out his wagon and drive over to Shelburne Falls for more whiskey. Just as he was about putting the finishing touches on the harnessing arrangement, he said to himself : "This horse has got horns !" He brought out his lantern, and found he had hitched the cow to the wagon. He muttered : "I'm drunk enough now," unhitched the beast, and went into the house to sleep it off.

### "I KNOW THAT."

A London paper has heard of a case where a droll fellow named Scrubbs got into a first-class railway carriage, before smoking carriages were invented. In the carriage was seated a sour-looking old gentleman. After the train had started, Scrubbs took out his pipe.

"You mustn't smoke here," at once said the old gentleman.

"I know that," replied Scrubbs. He then calmly filled his pipe.

"Did I not tell you," said the o. g. again, "that you can't smoke here?"

"I know that," gloomily replied Scrubbs, taking out his fusee box. He lit a fusee, but now the wrath of the o. g. was dreadful.

"You shan't smoke here, sir!" he shrieked.

"I know that," added Scrubbs, allowing the fusee to exhaust itself, when he lit another, and another; the stench was awful, the smoke suffocating.

"The o. g., coughing and spluttering, struggled for words. "You'd better smoke," said he.

"I know that," replied Scrubbs, applying the blazing fusee to the expectant pipe.

### A NEW WAY TO PROPOSE MARRIAGE.

The tide is turning at last. A young man in Nelson County, Iowa, armed himself with a revolver and sallied out to shoot a young woman who had declined the offer of his hand. But she was up to snuff. She read the papers and had frequently seen accounts of similar affairs, quietly resolving that no discarded lover could make a victim of her, not if the court, or rather the courted, understood herself. When the young man arrived at the house on his deadly mission he found the fair but cruel one in the kitchen doing the week's ironing. She didn't appear to suspect, and he expected to have an easy time preparing her for the coroner; but when he reached around to the pistol pocket, with the remark that her time had come, she stated, "I guess not," and knocked him down with the flatiron, demolishing his nose and front teeth. Then she gave him the scalding contents of a tea kettle that was singing a cheerful air on the stove, and when the family came in she was mopping the floor with him. The next time he proposes and is refused he will probably conclude that that settles it.—*Cincinnati Saturday Night.*

